# Green Physical Activity Indicator: Health, Physical Activity and Spending Time Outdoors Related to Residents Preference for Greenery

**DOI:** 10.3390/ijerph20021242

**Published:** 2023-01-10

**Authors:** Dagmara Stangierska, Beata Fornal-Pieniak, Paweł Szumigała, Katarzyna Widera, Barbara Żarska, Karolina Szumigała

**Affiliations:** 1 Department of Pomology and Horticulture Economics, Institute of Horticultural Sciences, Warsaw University of Life Sciences—SGGW, Nowoursynowska 166, 02-787 Warszawa, Poland; 2 Department of Environmental Protection and Dendrology, Institute of Horticultural Sciences, Warsaw University of Life Sciences—SGGW, Nowoursynowska 166, 02-787 Warsaw, Poland; 3 Department of Landscape Architecture, Faculty of Agronomy, Horticulture and Bioengineering, Poznań University of Life Sciences, ul. Dąbrowskiego 159, 60-594 Poznań, Poland; 4 Department of Economics, Finance, Regional and International Research, Faculty of Economics and Management, Opole University of Technology, Prószkowska 76, 45-758 Opole, Poland

**Keywords:** greenery, green spaces, feeling healthy, health declaration, physical activity

## Abstract

Spending time in the natural outdoor environment is a part of a healthy lifestyle. This study focused on identifying elements of green infrastructure that have a positive impact on both increasing physical activity, spending time outdoors and improving overall health. The aim of the research was to identify which elements of the settlement units’ green and blue infrastructure, related to residents’ preferences for greenery, influence more physical activity and spending time in green spaces and improve the healthy feeling of users as perceived by respondents. A total of 721 respondents from Poland took part in the survey. Using multiple regression models, the factors that influence an increase in outdoor physical activity Green Physical Activity Index (GPAI) were: using green spaces for exercise, spending time outdoors, exposure to nature and sufficiently large amounts of green space in the neighborhood and proximity to places to walk the dog. In contrast, physical activity has been shown to improve feeling healthy (health declaration). The main findings show that the increase in physical activity outdoors (GPAI) is positively influenced by factors related to respondents’ reasons for being outdoors, rather than the attractiveness and availability of green infrastructure. The research confirmed the necessity of arranging green areas with rich offerings in terms of a variety of activities for leisure visitors, to give them more opportunities for being outdoors.

## 1. Introduction

Green areas perform a lot of functions; in particular, they play an important role in the wellbeing of the residents, because these terrains are useful for various activities, including sports, as well as for shaping interpersonal relationships and social cohesion; all this together creates important influences on the “green lifestyle” of residents [1,2,3]. The relationship between urban and rural residents’ physical activity and green spaces has been a subject of interest for many scientific disciplines, including sociology, economics, urban planning and medicine [4,5]. Numerous studies have shown that physical activity improves quality of life [6,7,8].

The multi-dimensionality of this topic is reflected in the many concepts of healthy lifestyles and quality of life that have been formulated from the point of view of a particular science. For instance, in the field of economic science, the dependence of quality of life on economic conditions has been pointed out [9]. In the sociological sciences and psychology, on the other hand, there are references to the individuals’ subjective feelings to reality [10,11]. In medicine, the relationship between health status and the degree of daily physical activity is formulated; in urban and landscape planning, issues of physical activity are considered in terms of the functionality of areas [12,13,14]. Regardless of the scope of these definitions, physical inactivity is considered by the World Health Organization as one of four risk factors contributing to global mortality [15]. Numerous studies have shown that physical inactivity is associated with mental disorders, obesity and chronic diseases, such as type II diabetes and cardiovascular disease. Encouraging physical activity is therefore recognized as an effective strategy to prevent continuous increases in disease and healthcare expenditure [16,17].

Spending time in the natural environment is a part of a healthy lifestyle. Increasing time spent outdoors can result in a more active lifestyle and lower chronic disease risk [18]. Studies have shown that being outdoors (active and passive) lowers blood pressure [19]. In turn, forest bathing has been positively associated with human immune function [20]. In addition, research by the University of New England showed the positive effects of exposure to greenery on diseases, such as reductions in the risk of type II diabetes, cardiovascular disease, premature death, preterm birth, stress and high blood pressure [21].

Research has also indicated that the availability of green spaces promotes physical activity, contributing to lower levels of obesity [22]. Just being able to see greenery has a documented positive effect on the faster recovery of patients after surgery [23]. 

An important aspect of a person’s overall wellbeing is mental wellbeing, which not only determines the general state of health, but, to a significant degree, determines the physical condition of the whole organism. Increasingly, it is the difficulties of everyday life that trigger the body’s defensive reactions in the form of tension and stress, which have a negative effect on a person’s mental and physical health. [13]. Contact with the elements of green spaces causes regenerative reactions, and the scenery that gives rise to such reactions is called a regenerative environment [24]. The perception of the surrounding reality is conditioned by the biological memory of the past. The positive influence of nature on man is due to his past and the fact that man is a part of nature [25,26]. Green spaces can act as a buffer against the negative health effects of stressful life events [27,28,29]. Spending time in natural outdoor environments is a part of a healthy lifestyle; depending on the source, a minimum of 20–30 min a day is considered necessary for the amount of time spent outdoors [30,31,32].

Due to the extensively documented relationship between health and green spaces, it is now even more important to realize the concept of sustainability in the design of urban areas, including the contribution of green spaces and water [33,34]. In this context, the quality and accessibility of green and blue areas are not just an aesthetic requirement. The contribution of green spaces and surface water to urban land use has an increasing impact on raising the urban and landscape standards of these areas [35]. Green areas, including water, are elements of the environment that also have an impact on the economic field of urban investments, and, in the context of climate change, dynamic civilizational development, and are helpful against new threats (e.g., epidemics, COVID-19); green areas are becoming almost a necessary condition for urban existence [36,37].

The availability and standards of urban green spaces are of increasing significance for human health. Through dedicated landscaping of urban green spaces and recreation areas, we can stimulate the human senses to a great extent and induce a positive effect on the human body and mind. Through dedicated landscaping of urban green spaces and recreation areas, we can stimulate people’s senses to a great extent and evoke reactions that are desirable from the point of view of their health [38].

As the impact of both physical activity and the outdoors is so important from a preventive health perspective, research to date has primarily focused on the relationship between outdoor time and physical activity [39,40,41,42]. In our study, we focused on the identification of green infrastructure elements that have a positive impact on increasing physical activity, spending time outdoors and improving general health.

The object of this research was the link between residents’ various physical activities (including sporting activity) and green spaces as a convenient place for this purpose. The different types of sporting activity were jointly treated as a sporting activity—one from many types of physical activity. The intention was to find out whether respondents practice various activities in green areas and, depending on the type/level of a preferred activity, what attributes of green areas they pay attention to. The aim of the survey was to identify which elements of the settlement units’ green and blue infrastructure (green areas), related to residents’ preferences for greenery, influence more physical activity and spending time in green areas, as well as users feeling healthier, as perceived by respondents.

**H1.**  *The level of physical activity of the respondents significantly determines the reasons for using green spaces in the settlement unit, the overall evaluation of green spaces, as well as the importance of the individual elements in them, depending on the preferences of the respondents.*

**H2.**  *Respondents’ evaluation of their health is positively correlated with their physical activity, for which green areas are very useful; this subjective evaluation of their health depends on the frequency and duration of their activity in green areas, on the presence of preferred natural elements that form green areas and on their overall satisfaction with the green areas in their place of residence.*

## 2. Materials and Methods

### 2.1. Study Design 

**Figure 1:**
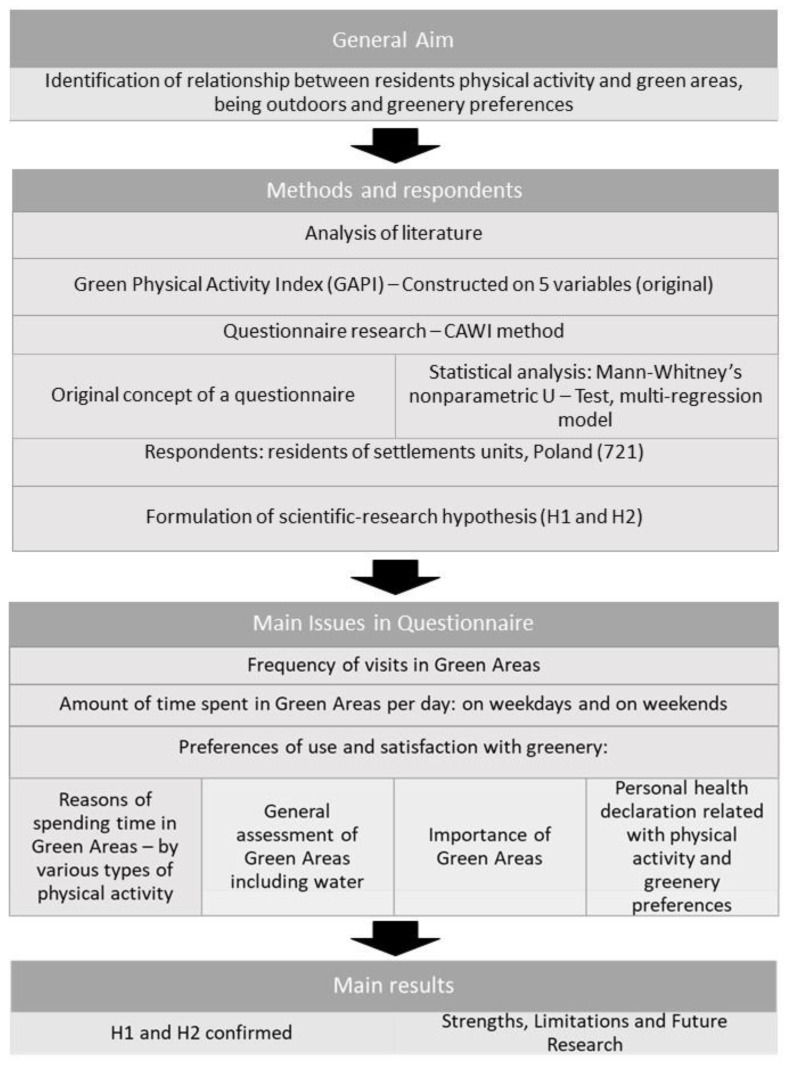
Framework of the study.

The paper was based on the results of a questionnaire research study conducted in 2022 using the CAWI method. A brief description of the study and its purpose, as well as a declaration of anonymity and confidentiality, were given to participants before they started the questionnaire. Respondents did not provide their names or contact details (including IP addresses) and could terminate the survey at any stage if their questionnaires were not saved. The online survey was conducted in full respect of national and international laws in line with the Declaration of Helsinki (2000). Participants’ information and personal data were anonymized in accordance with the European Parliament’s General Data Protection Regulation (GDPR 679/2016). Participation in the study was entirely voluntary. Study participants were recruited among people registered by personal contact and social media. Such research did not require the approval of any ethics committee. A total of 721 people took part in the survey (Figure 1). 

### 2.2. Questionnaire

The research questionnaire consisted of three main parts. 

The first part of the questionnaire contained questions regarding respondents’ characteristics, accounting for gender, age, place of residence, education and level of income.

The second part of the survey included two subsections: quantitative use of greenery and qualitative aspects of greenery preferences. In the first subsection, respondents answered three questions regarding the frequency and amount of time spent surrounded by green.

- Frequency of visits to green areas was measured on a five-point interval scale: 1 for less than once a week; 2 for 1–2 times a week; 3 for 3–4 times a week; 4 for 5–6 times a week; 5 for every day.
- The next two questions regarded the amount of time per day spent on average in a green environment, distinguishing between weekdays and weekends, using a five-point interval scale: 1—not at all; 2—less than 20 min; 3—for 20–60 min; 4—for 1–2 h; 5—for 2 h or more. A time limit of 20 min was set for the correct and incorrect amount of time spent, as according to Hunter et al. [32].
- In order to determine preferences related to the use of and satisfaction with greenery, questions were asked regarding three aspects: reasons for spending time in green areas were answered from 1—totally disagree to 5—totally agree. The response reasons consisted of: walking; leisure; exercise and sports; being out; spending time with loved ones; communing with nature; health reasons.
- Questions regarding the general assessment of green areas in a settlement unit of residence was measured by using the seven-item scale proposed by Pope et al. [43] and referred to whether green spaces were localized, including: accessible; well kept; sufficient and within walking distance; whether these green areas were perceived as relaxing ones or possible to use for recreation. The questions had a three-category response option, including: “Agree”, “Disagree” or “Not Applicable”.

The importance of green elements in a place of residence was measured with a five-point scale from 1—very unimportant to 5—very important, concerning important sites: shared courtyard, playground, safe place where children can play, proximity to large parks and proximity to squares where people can sit and relax, green recreational areas where people can play sports, proximity to places where people can walk their dogs, green belts between the pavement and the street, small ornamental trees by the streets, large spreading trees by the streets, short distance to the forest and water elements in green areas. 

In the last section, three questions were used from the Beliefs and Eating Habits Questionnaire (KomPAN) on physical activity on weekdays and weekends, and four questions regarding personal health declaration compared to peers [44] were measured using three statements corresponding to a high level, average level and low level.

### 2.3. Characteristics of Respondents

In terms of gender, the sample consisted of 65% women and 35% men. The age structure of the studied group was: 18–25 years old—54%; 26–35 years old—16%; 36–55 years old—22%; 55 and older—8%. In terms of education, over half of respondents were people with higher education (51.87%); a total of 41.5% of the respondents had completed secondary education, 5.85% had vocational education and only 1.8% had completed primary education. Nearly 50% of the respondents lived in cities with a population larger than 200,000 people; the second biggest group were rural residents—24.27%, 15% lived in towns of under 50,000 people and 13.2 % in towns with 50,000–200,000 residents. Analysis of the economic situation of the respondents showed that over 40% of them had a monthly income of PLN 1501–3500 per person, while 25.6% had an income in the range of PLN 3501–4500; a total of 14.98% of the respondents had an income over PLN 5500, and the smallest group of respondents earned less than PLN 1500 per person per month (Table 1). 

### 2.4. Green Physical Activity Indicator

In order to study the phenomenon of physical activity outdoors, the Green Physical Activity Indicator (GPAI) was constructed. As this can be measured by a number of characteristics, five variables were chosen to construct this indicator: frequency of visits to green areas; amount of time per day spent on average in a green environment on weekdays; amount of time per day spent on average in a green environment at weekends; physical activity on weekdays and physical activity at weekends. Once the initial indicator values were determined, they were shifted on the scale by subtracting 4. The final Green Physical Activity Indicator (GPAI) values were taken from 1 to 11, where 1 was the lowest indicator level and 11 was the highest. 

### 2.5. Statistical Analysis

On the basis of the data obtained in the empirical study, variables were identified on the basis of which the research hypotheses were verified. These variables were measured on a rank scale, which determined the use of appropriate statistical tools. For all hypotheses in the statistical analysis, a significance level of α = 0.05 was adopted. The use of Mann Whitney’s non-parametric U-test allowed for the determination of statistical significance of differences, or lack thereof, in preferences related to the use of and satisfaction with greenery (reasons of spending time in green areas, the general assessment of green areas, importance of green elements in the place of residence) between pairs of physical activity groups on weekdays and weekends.

The constructed Green Physical Activity Indicator (GPAI) and greenery preferences were used as explanatory variables in a multiple regression model. Based on the estimated model given by Equation (1), variables were identified that significantly showed a cause-effect relationship between the GPAI and greenery preference by respondents. The proposed model showed good verification properties, as analysis of the distribution of its residuals indicated that their distribution was consistent with a normal distribution. This was confirmed by the value of Pearson’s χ^2^ statistic = 9.8949 and the corresponding *p*-value = 0.3591 > 0.05.

The next step in the statistical analysis was the construction of a second multivariate regression model, which looked for determinants of health declaration in relation to physical activity and green space preference. This model, given Equation (2), also showed that the distribution of its residuals was consistent with a normal distribution. The value of Pearson’s χ^2^ statistic = 7.49314 and the corresponding *p*-value = 0.05773 > 0.05 confirmed the verification properties of the proposed model. By verification properties, we refer to the validity of the model as a tool to verify the proposed research hypotheses.

All calculations were made using Statistica 14.1 program.

## 3. Results

### 3.1. Level of Physical Activity and Preference for Green Space

When comparing the reasons for using green spaces between people with low and moderate physical activity, regardless of whether the activity was determined for weekdays or the weekend, people with lower physical activity showed less importance for all reasons for using green spaces compared to people with moderate physical activity. However, in the case of weekday activity, the changes were only statistically significant for two reasons, people with lower physical activity compared to people with moderate physical activity visited green spaces significantly less often for health reasons and to actively spend time. In the case of physical activity only declared for weekend days, the differences were not statistically significant for visiting green spaces to spend time with family. Comparing the reasons for visiting green spaces between the most complainant groups of low and high physical activity, in the case of weekdays, those with low activity were statistically significantly more likely to visit green spaces for walking and relaxation. At weekends, people with higher physical activity were statistically significantly more likely to visit green spaces for exercise (the highest difference of all analyzed groups), to commune with nature, to spend time outdoors, to relax and for health reasons. The least differences were observed when comparing the reasons for using green areas between people with moderate and high physical activity; in the case of the level of activity declared on weekdays, people with moderate physical activity declared significantly more often visiting green areas for walking and relaxing, while on weekend days people with high physical activity declared significantly more often visiting green areas for actively spending time (Table 2). 

When comparing the attributes of green spaces between groups of people with different levels of activity, there were only a few statistically significant differences between the low and high activity groups at the weekend. People highly active at the weekend compared to moderately active people also paid significantly more attention to a sufficient amount of green space and the possibility to relax (Table 3).

In the case of the importance of green space elements, the most statistically significant differences were found between those with the lowest and highest physical activity. In the case of activity on weekdays, the following were significantly more important for people with low activity: proximity to large parks, green belts between the pavement and the street and large spreading trees by the streets. In the case of declared activity on weekend days, people with high physical activity compared to people with low physical activity attached statistically significantly more importance to a shared courtyard, playground, safe place where children can play and green recreational areas where people can play sports. People with lower physical activity compared to moderate physical activity at weekends placed significantly less emphasis on shared courtyards, playgrounds, safe places where children can play, green recreational areas where people can play sports and proximity to places where people can walk their dogs. People with moderate physical activity on weekdays compared to people with high activity considered proximity to large parks and green belts between the pavement and the street to be statistically significantly more important (Table 4).

### 3.2. Green Physical Activity Indicator and Greenery Preferences

First multiple regression model describing relationship between the Green Physical Activity Indicator (Y_GPAI_) and preference variables (X1–X_5_) related to the reuse of and satisfaction with greenery (described in the table) is given by Equation (1).

(1) $$Y_{\mathrm{GPAI}}=2.104+0.477\cdot X_{1}+0.161\cdot X_{2}+0.183\cdot X_{3}+0.280\cdot X_{4}-0.159\cdot X_{5}+0.162\cdot X_{6}+e$$

All structural parameters next to the explanatory variables Xi in the model given by Equation (1) are statistically significant, as all p-values in the table are less than the significance level α = 0.05 assumed in the study (Table 5). As the importance of using green spaces for exercise (X_1_), being outdoors (X_2_), exposure to nature (X_3_), appropriately large amounts of greenery in my neighborhood (X_4_) and closeness to places where you can walk your dog (X_6_) increases, the value of the Green Physical Activity Indicator of respondents (Y_GPAI)_) increases. In contrast, the greater the proximity to squares where you can sit and relax (X_5_) the lower the Y_GPAI_ value (Table 5).

### 3.3. Health Declaration, Phisical Activity and Greenery Preferences

The second multiple regression model was designed to represent the relationship between the evaluation of health declaration (hd) (Y_HD_) (and variables (X_7_–X_11_) related to the use of and satisfaction with greenery and physical activity described in the table) is given by Equation (1).

(2) $$Y_{\mathrm{HD}}=0.990-0.039\cdot X_{7}+0.041\cdot X_{8}+0.127\cdot X_{9}+0.130\cdot X_{10}+0.291\cdot X_{11}+u$$

For each structural parameter in model (2), the condition of statistical significance was found to be met, as all p-values in the table are less than the significance level α = 0.05 assumed in the study (Table 6).

As the importance of using green spaces for walking increases (X_7_), the respondent’s health declaration score decreases. The greater the respondents’ use of green spaces to exercise (X_8_), their rating of green spaces as accessible (X_9_) and their physical activity during the week (X_10_) and at the weekend (X_11_) is greater; the higher the respondent’s health declaration score (Y_HD_). The model shows an analogous relationship of an increase in respondents’ health declaration rating with an increase in physical activity during weekdays and at weekends (Table 6).

## 4. Discussion

The results show that people with higher reported physical activity have more reasons for using green spaces, particularly those related to spending time being active (H1). This is consistent with research by Sugiyama et al., 2013 [45], who determined that specific types of physical activity, such as walking, are associated with certain UGS characteristics, such as accessibility to green space and total neighborhood green space. Similar findings were taken from research conducted in England, that there is a link between health outcomes and green spaces; due to increased levels of physical activity of individuals living in areas with more green space, but association was found between green space and types of physical activity normally associated with green space. In turn, research by Akpinar et al. [46] showed that green infrastructure elements, such as the number of trees, exercise equipment and playing fields for team sports, are positively associated with physical activity.

Research has shown that there is a significant relationship between the amount of green space and increased physical activity among residents. This relationship has been demonstrated in studies [47] and research has been conducted in eight European cities, as well as cities on other continents [2,48]. In addition, the research by García de Jalón et al. [49] indicated that increased availability of green spaces is found to be associated with a reduction in sedentary time and an increase in walking; and research by Akpinar et al. [46] showed short distance to urban green spaces was positively associated with the frequency of physical activity. Our research indicated that the perceived accessibility of green spaces is more important than their amount, and the higher the physical activity, the more residents believe that green spaces are available in their place of residence.

Other research has shown that people living in the greenest areas of England are more likely to achieve the recommended levels of physical activity [48,49]. Our study found that people with high levels of physical activity depend on the proximity of green spaces from where they live and frequently use these spaces (H1). 

The impact of urban green spaces on people’s likelihood to engage in physical activity varies depending on the type of activity. This may explain why some studies have shown that green spaces “encourage”, e.g., physical activity through appropriate landscaping, which translates into a reduced likelihood of overweight respondents [50,51]. In contrast, if green spaces were otherwise spatially managed contrary to users’ expectations, no positive relationship was found in this aspect [49,52], which coincides with confirmed hypothesis H2.

In contrast, another study [53] shows that the amount of green space in a residential environment is weakly related to physical activity levels. Furthermore, the amount of physical activity undertaken in an area with more green space does not explain the relationship between green space and health.

The results obtained from our study depict that physically active people use green spaces that are then “appreciated” by them (verified H1 and H2). In contrast, increasing the number of green spaces does not increase the number of physically active people (who were not physically active before), as shown by previous research [53].

The results of another study [54] showed that the time to reach green spaces and also the types of green spaces respondents could use for physical activity were significantly related to satisfaction levels. Those who could use all public green spaces and parks for physical activity reported higher levels of satisfaction. The less time a respondent needed to reach a park, for example, the higher the level of self-reported satisfaction regarding their health declaration, which is in line with our findings (H1). This is the case when neighborhood green spaces are too small to support physical activity. Studies showing that residential proximity and physically active people are strongly associated with physical activity and use of green spaces in cities, as shown by Liu et al. [54], and these associations were also confirmed in our study (H1 and H2). Physical activity in parks was significantly associated with mental health benefits and physical activity [54].

Green spaces are places where people can improve their health through physical activity. Increased activity in urban green spaces has benefits for the self-assessed health declaration of city dwellers, as confirmed in our study by respondents who were physically active (H2). Our findings are consistent with the results of a meta-analysis performed in [4], which concluded that green spaces improve human physical health and wellbeing by providing space for exercise, jogging, walking, cycling and other recreational activities. Other researchers have found that reductions in cardiovascular disease, being overweight, poor general health and poor mental health in New Zealand have been linked to green spaces through physical activity [55]; in California, children living within 500 m of parks have been shown to be more active and less likely to be obese [56]. Results from the U.S. [57] showed that greater exposure to trees in daily life is associated with better health outcomes. Specifically, higher neighborhood concentrations of tree canopy are related to better physical health, overall health and an increased capacity to control stress. 

## 5. Strengths, Limitations and Future Research

A strength of this study is the innovative methodological element of the research tools—the original concept of a questionnaire to recognize respondents’ opinions, which featured: multi-faceted construction, special selection of topics for questions, sequence of questions, etc., while maintaining a compact design, which has allowed us to investigate the more complex issue of the relationship between physical activity, health status and green space characteristics. This approach allowed us to develop a Green Physical Activity Indicator (GPAI) which, in the light of obtained results, may be useful in future studies.

The research study was undoubtedly limited by the fact that the sample consisted exclusively of Polish consumers, which calls for confirming the observed correlations with studies in other countries. The main limitations of our article are due to the specifics of survey research, the drawbacks of which include subjectivity, recall bias, reporting errors and individual preferences

It would also be advisable to detail future studies with aspects such as finding out preferences in terms of the types of sports activities that residents practice in green spaces and how these are linked to the green space program. In contrast to the completed survey, it would be useful to explore both health and mental health as variables associated with outdoor activity in more depth.

## 6. Conclusions

The results of the study fully confirm the first hypothesis (H1) in the context of the differentiation of reasons for using green spaces depending on the level of physical activity and the importance of individual elements of green infrastructure for the respondents. It was not confirmed that the overall assessment of green spaces depended on the level of physical activity. The second hypothesis (H2) of the study was fully confirmed, allowing us to conclude that a sense of health is dependent both on the level of physical activity, spending time outdoors and the availability of and reasons for using green spaces.

The results show that people with varying levels of physical activity show numerous differences in the importance of reasons for using green spaces. The higher the activity level, the greater the importance of spending time actively outdoors. Green spaces are equally attractive to people with different levels of physical activity. At the same time, people with higher activity levels find the additional aspects of green spaces that enable them to actively spend time more important, while people with lower activity levels pay attention to the proximity of infrastructure and its recreational value. The research confirmed the necessity of arranging green areas with rich offerings in terms of a variety of activities for leisure visitors, and confirmed the necessity of protecting the remnants of nature in the settlement units and their close vicinity. The necessity is firmly embedded in the consciousness of the inhabitants of settlement units (towns and villages), so it is worth maximizing the quality of green and blue infrastructure in the planning of settlement areas. 

Multiple regression analyses showed that an increase in outdoor physical activity (GPAI) is positively influenced by factors related to respondents’ reasons for being outdoors, rather than the attractiveness and availability of green infrastructure. Previous research has indicated that increasing the availability of green space helps to improve health and physical activity. Our research indicates that assessment of the availability of green spaces does not vary with activity levels and, therefore, promoting active outdoor pursuits is a sensible direction. Information and promotion of outdoor activities should be a part of preventive health protection to a greater extent than simply developing green infrastructure. This is particularly important, as subsequent studies have shown that declared health was most influenced by physical activity and outdoor exercise.

Future studies could deepen the analysis in terms of factors related to both green space chemistry and health education to increase outdoor activity, including specific forms of activity and how to develop the best tools to promote activity in green spaces.

Our research results are well correlated with the future vision of European cities. According to the expertise of the European Commission [58], future cities should have such features, among others, as: being ecological and healthy, possessing a lot of green areas including natural ecosystems, green areas being a place of social relationship and cohesion; and, in the variant of the Green City of the Future, these characteristics are highly enhanced, as: (1) urban areas are to be a vast network of greenery and water, (2) greenery on every possible site (green roofs, walls, balconies), (3) ecological life of residents and (4) land recycling (direction of change: mainly green areas).

## Figures and Tables

**Table 1:** Characteristics of the study population (n = 721, data in %).

Gender				
Female		Male		
64.77		35.23		
Age				
18–25	26–35	36–55	Over 55 years old	
53.54	16.37	21.91	8.18	
Education				
Primary	Vocational	Secondary	Higher	
1.81	4.85	41.47	51.87	
Residence				
Village	City with up to 50,000 inhabitants	City with 50,000 to 200,000 inhabitants	City with more than 200,000 inhabitants	
24.27	15.26	13.18	47.29	
Per Capita Income PLN/EUR *				
Under 1500 PLN (320.14 EUR)	1501–3500 PLN (320.15–746.98 EUR)	3501–5500 PLN (746.99–1173.83 EUR)	Over 5500 PLN (over 1173.84 EUR)	no answer
11.10	43.13	25.80	14.98	4.99

* *p* < 0.05. As of 5 December 2022 NBP Exchange rates are Available online: https://www.nbp.pl/home.aspx?f=/statystyka/kursy.html, accessed on 11 December 2022.

**Table 2:** Reasons for using green spaces comparison of people with different activity levels (pairwise comparisons using the Mann-Whitney U-value Z-test).

Reasons	PA Low vs. PA Medium		PA Low vs. PA High		PA Medium vs. PA High	
	Weekdays	Weekends	Weekdays	Weekends	Weekdays	Weekends
Walking	−0.622	−2.493 *	3.204 *	−1.808	3.347 *	−0.148
Relaxing	−1.713	−3.113 *	1.985 *	−2.414 *	2.971 *	−0.082
Being outside	−1.319	−2.615 *	−0.431	−3.292 *	0.430	−1.455
Spending time with family and friends	−0.543	−1.539	0.216	−0.653	0.518	0.580
Exposure to nature	−1.030	−2.812 *	−0.252	−3.520 *	0.349	−1.536
Health reasons	−2.008 *	−3.132 *	−0.452	−2.635 *	0.823	−0.324
Exercise, sport	−3.954 *	−6.818 *	−1.104	−8.573 *	1.336	−4.680 *

* *p* < 0.05.

**Table 3:** Attributes of green space comparison of people with different activity levels (pairwise comparisons using the Mann-Whitney U-value Z-test).

Attribute of Green Space	PA Low vs. PA Medium		PA Low vs. PA High		PA Medium vs. PA High	
	Weekdays	Weekends	Weekdays	Weekends	Weekdays	Weekends
Accessible	−0.780	0.344	0.263	0.106	0.761	−0.170
Well kept	0.279	0.794	1.340	0.102	1.063	−0.549
Safe	−0.996	−0.920	0.316	−0.304	0.940	0.432
Can relax in green space	−0.428	0.613	0.409	−1.588	0.665	−2.148 *
Can use for recreation	−1.629	−1.864	−0.229	−2.430 *	0.825	−1.127
Within walking distance	0.016	−0.798	0.443	−0.119	0.403	0.550
Sufficient in neighborhood	−1.449	−0.030	−0.608	−2.549 *	0.362	−2.630 *

* *p* < 0.05.

**Table 4:** Importance of green elements comparison of people with different activity levels (pairwise comparisons using the Mann-Whitney U-value Z-test).

Importance of Green Elements	PA Low vs. PA Medium		PA Low vs. PA High		PA Medium vs. PA High	
	Weekdays	Weekends	Weekdays	Weekends	Weekdays	Weekends
Shared courtyard, playground, safe place where children can play	−0.933	−2.412 *	−0.675	−2.121 *	−0.127	−0.280
Proximity to large parks	0.412	−0.882	2.854 *	0.023	2.357 *	0.757
Proximity to squares where people can sit and relax	1.810	0.573	1.683	1.820	0.545	1.539
Green recreational areas where people can play sports	−1.156	−4.988 *	1.310	−4.331 *	1.883	−0.738
Proximity to places where people can walk their dogs	−1.631	−2.033 *	−0.087	−0.859	0.947	0.716
Green belts between the pavement and the street	−0.996	−1.135	2.063 *	0.582	2.480 *	1.494
Small ornamental trees by the streets	0.337	−0.364	−0.157	1.415	−0.417	1.811
Large spreading trees by the streets	0.777	−0.878	2.225 *	−0.853	1.579	−0.252
Short distance to the forest	−0.259	−2.312 *	1.118	−2.352 *	1.250	−0.637
Water elements in green areas	0.136	−0.715	0.327	−0.666	0.197	−0.155

* *p* < 0.05.

**Table 5:** Parameter values of the multiple regression model describing the relationship between the Green Physical Activity Indicator and greenery preference and the corresponding *p*-values.

Variable		Evaluation b	*p*-Value
Exercise, sports (reasons)	X_1_	0.477	0.000
Being outside (reasons)	X_2_	0.161	0.027
Exposure to nature (reasons)	X_3_	0.183	0.008
Sufficient in neighborhood (attribute of green space)	X_4_	0.280	0.001
Proximity to squares where people can sit and relax (importance of green elements)	X_5_	−0.159	0.048
Proximity to places where people can walk their dogs (importance of green elements)	X_6_	0.162	0.004
Constant		2.104	0.000

**Table 6:** Parameter values of the multiple regression model describing the relationship between health declaration Green and Physical Activity and greenery preferences and the corresponding *p*-values.

Variable		Evaluation b	*p*-Value
Walking (reasons)	X_7_	−0.039	0.040
Exercise, sports (reasons)	X_8_	0.041	0.012
Accessible (attribute of green space)	X_9_	0.127	0.001
Physical activity on weekdays	X_10_	0.130	0.000
Physical activity on weekends	X_11_	0.291	0.000
Constant		0.990	0.000

## Data Availability

The authors confirm that the datasets analyzed during the study are available from the corresponding author upon reasonable request.
